# Proceedings of the Annual Meeting of the A. S. D. S.

**Published:** 1852-04

**Authors:** 


### Selections.

### THE PROCEEDINGS

*Of the Twelfth Annual Meeting of the American Society of Dental Surgeons, held in Philadelphia on the* 5*th*, 6*th*, *and* 7*th of August*, 1851.

[Continued from page 117.]

Dr. Cone.—The question arises, whether the teeth of patients, under all circumstances of age and pathological conditions, should be equally treated, and if so, will there be equal success? Is there not a difference? Is a tooth in a young patient treated with the same degree of success and permanency, as in a patient of middle age? Has the previous condition of the health of the tooth anything to do with the permanency or success of the operation? Also, has the relative condition of the tooth with its neighbors anything to do with it? These thoughts I throw out to get the opinion of the members present.

Dr. Westcott.—Mr. Chairman, I must say I am glad this particular subject has been selected for our discussion this morning; not merely from its importance, but from the fact that there has been a great deal of discordant testimony upon it by different and by the same individuals. I happen to be one of these same men. It is well known to many of you, that this is a subject upon which I had formerly written somewhat. Since then my views have been somewhat modified; and these convictions have arisen, not merely from greater experience on my part, but more from the enlightened experience of my friends, to whom I am much indebted for such information, Dr. Maynard particularly. Yet, with all that I have been enabled to gather, both from the experience of others and my own, I confess that I look upon the operation of destroying nerves with arsenic, not with that same degree of certainty that Dr. Dunning has expressed. The plan I then proposed was the excision, which has been talked of as a substitute; and I now say, that, if we could but lay aside the painfulness of the operation, I prefer it by far to any other method of destroying the nerve. I think that if a live nerve is cut off at its upper end, the remaining portion has the power of taking care of itself. I do occasionally, and quite frequently—seemingly from being absolutely compelled to do so—employ arsenic or cobalt to destroy the nerve, but I confess never with the same success as by the other method. I wish to make a general remark upon the subject, with reference to the first two aphorisms. It occurs to me that they might be put into one, and could cover all that we are authorized to cover. We can do little but give our respective practices. For me to say that nerves should never be destroyed, would not be right; and for another to say they should, I can hardly agree to. The truth is, that every man's practice is thoroughly modified by the circumstances peculiar to his own practice, though done upon general principles, that I have long since ceased to condemn processes. Give me the result; let the result come out right, and I will accord to every man his own process. For instance, Dr. Dunning's practice is made up of the first class of patients in New York—persons, without exception, that take the utmost care of their teeth. He probably does not perform an operation on a single individual who does not care for his teeth with the utmost scrupulousness. This cannot be so in a country practice. I practice in a small city, but my practice, in the one-half, is made up of persons in the surrounding country. For me to take the position that I would not operate on any one who did not take such care of his teeth as I should dictate, would be to cut off one-half of my practice, though in some extreme cases I have done it. My practice is very much modified by this circumstance—different from what Dr. Dunning's practice would be. I then feel that about all we can do upon this occasion is for each to give his own practice, and the reasons for it; and I would be glad if the aphorism should be so worded as to admit of the question being an open one hereafter. Nevertheless, opinions and practices might be so given as to serve as a strong guide to the young practitioner.

One of these aphorisms reads that a tooth may be permanently saved when the nerve is exposed, and another that the vitality of the pulp must be destroyed. We could make them read in this way: A tooth, whose nerve is exposed, may be successfully saved. And if these aphorisms are intended to bear particularly upon the destruction of the pulp, it may read simply—"successfully saved by the detruction of the pulp."

In respect to Dr. Foster's testimony, I don't remember precisely when Dr. Dunning and myself commenced this. It must be eight or nine years ago. We treated mutually and jointly, all our cases, being in the same office. We treated with the probe, and I must say that, so far as my experience has gone since that period—and it is proper here to observe that Dr.

Dunning and myself diverge—I have found the use of the probe not merely practicable, and practicable upon timid patients, but I have to say, so far as ultimate results are concerned, it was far more certain of warding off future inflammation. My practice has been to remove the nerve and at once to plug the tooth with gold with a delicate instrument, which shall go as far as possible to the point where the nerve has been excised; and when I do this in my own way, I have very little fear of inflammation supervening in a healthy mouth that is taken care of. But I do resort--seemingly from necessity, and that necessity based upon the feelings of the patient—to the use of arsenic, in some way or other, to destroy the nerve, and frequently with tolerable success—it being true that the result of these operations depends much on extrinsic circumstances, which the dentist cannot control.

In regard to capping nerves, I confess I never had any success in it. This may be humiliating—it may be tantamount to saying that I do not know how to do it; but I have tried it, and have not been successful. It is something that is important in the extreme, and I shall be most thankful to any gentleman who will so place it before my mind that I may be successful hereafter on the capping system—that is, leaving the nerve entire.

I close by observing that if these first two aphorisms could be so shaped as to include the fact that a tooth might be successfully treated, for I should not be safe in using the word permanently, as it might give a false impression to the public, —I think the word permanently should be supplied by the word successfully, and then so shape them as not to convey the idea that this is the only way in which exposed nerves may be treated, leaving the other an open question—leaving us to accumulate all the light we can. If we can save nerves,—I think they were made for a good purpose,—let us do it. In Comb's Constitution there is a good illustration which shows the necessity of having nerves, and I think they are a very good accompaniment to the human system; and if we can save them in their normal condition, I feel that it is an important thing. I rose in reference to these two aphorisms, and I wish that they may be so shaped that they need not necessarily carry the idea that this is the only method of accomplishing the end.

Dr. Dunning.—In regard to the aphorisms, I would remark, that I consider them entirely the property of the society, and that they have been written in such haste that, on the contrary from feeling any pride in the construction of them, I feel a desire that the society should remodel them entirely, for I see many points, myself, that need it; and I hope that members will not feel the slightest hesitation in proposing any change that is required. In two places in Dr. Westcott's remarks, he refers to the nature of the practice of different individuals, as affecting the operation; but, with regard to my own practice, I wish to say that it is not among those whom I regularly attend, and who take the best care of their teeth, that I am called upon to perform this operation. It is often the case that I am obliged to remove decayed teeth and roots, that I think should be taken from the mouth, to bring it into a proper state. I think I have not, in the aphorism, properly illustrated my own views. I prefer, myself, the surgical operation, whenever it can be used. I have universally practised it in the incisors and very commonly in bicuspides, and sometimes in the molars. With regard to the capping system, I have not practised it, because I dare not. I have no doubt, from the testimony I have received, that it is successfully performed; and I think it is the highest success of the art. But I have seen many cases where the operation has been performed, and the nerve has evidently died, perhaps without even the patient's being aware of it, and the tooth has been left in a position in which it is very difficult to save it comfortably to the patient; and for this reason I have been afraid to do it.

Dr. Bridges.—I was very much pleased to hear some of Dr. Westcott's remarks, because it gave me an opportunity of saying a word. About nine years ago this arsenic question came up, and my friend Dr. Westcott said, in my hearing, he considered it a matter of humbuggery, and the man a humbug who used arsenic for killing the nerve of the tooth. He said he considered it malpractice. Since that time I have taken another view of the subject, and yet I have frequently used arsenic, and sometimes a red hot iron, with great success. After the condemnation of arsenic by most of the prominent members of the society, I had a conversation with Dr. Hill, and he and I concluded that mere cologne or alcohol applied to the nerve, for an hour or two, would so paralyse it that we could fill the tooth; and I practised that in ten cases out of twelve. In applying the hot iron, I found that if it cooled any it produced inflammation, but if it was red hot I most invariably met with success. I do not recommend this practice; but the other way of applying alcohol or cologne I have had very good success in. I should like to know if others, who have applied the same remedy, have been successful. I was very much delighted that Dr. Westcott gave me an opportunity of explaining this thing, for I felt much hurt at the time, and since then I find that he has changed his views on the subject of arsenic. And I heard you say once, Mr. President, that you would put down the man who used arsenic with the man who used amalgam.

I think in some cases arsenic can be used with great success where the pain is very acute and the nerve has been exposed for a long time. It is a very painful operation to excise the nerve, and in that case I would recommend an application of cotton, and afterwards moisten it with kreosote, and then put soft wax over it and let it remain from four hours to a day, and the nerve may bleed whenever you touch it, but the feeling is so far gone that you can do it with little pain. Dr. Westcott said that when he excised the nerve, there was very little danger of inflammation supervening in the remaining part. I would wish to know what is meant by the remaining part.

Dr. Westcott.—I mean that the nerve stops somewhere and connects with the main branch, and if not cut off at the root of the tooth, it is as near that as possible, though not to it.

Dr. Bridges.—I would like to hear what Dr. Hill has to say about the application of cologne and alcohol.

Dr. Hill.—I had forgotten the circumstances till they were mentioned, but I now remember them. Many years ago, before this subject was discussed among dentists, and when I was, like most other gentlemen practicing the profession, in the dark in this matter,—left to follow my own judgment as best I might with the light I could gather,—I had, like the rest of them, no definite idea of the mode of practice, and no distinct and certain knowledge as to the results that might follow an operation of this kind. I remember that, years ago, in a case of that kind, I would uncap the nerve so that it would bleed—I would excavate all around the nerve as well as I could, and leave the nerve as slightly touched as the nerve would allow; and very often I would find it bleeding, and I would take a small instrument and make it bleed, and if there was sensibility, the most convenient thing I could use was a little cologne or alcohol, and sometimes I have used tinct. of myrrh—and after wiping the cavity dry, put in my filling. In treating them in that way, a large proportion have been successful, and have been saved for a number of years; but I have noticed in some cases that suppuration has taken place. I early imbibed a dislike to the practice of using arsenic. When the subject was first presented to me, it struck me unfavorably. I did not doubt that in some cases it would successfully destroy the nerve, so that the tooth might be filled, but it seemed to me to be a dangerous practice, and I have used it in but very few instances. My ordinary treatment is that described by Dr. Foster. My preference is for that mode, decidedly. I have no doubt in my own mind that, in many cases, the nerve may be capped successfully. My own experience has demonstrated it to me; for though I did not operate upon that principle, yet I made the nerve bleed and let it contract, and then I could save the tooth—and it was virtually done by capping, though capping was little known then. In most cases, however, I destroy the nerve in the manner described by Dr. Westcott.

The President.—An explanation is necessary of the remark made by me, which has been alluded to by Dr. Bridges. I

have as strong an objection to the use of arsenic to-day, in the way it was then used, as I had on the day the remark was made. I opposed it strongly, as it was used to destroy the sensibility of the tooth, leaving the nerve in a paralyzed condition; and it generally resulted in suppuration and loss of the tooth. But it is used now to paralyze the nerve, so that it may be successfully used afterwards. It is thus I presume, that all who are successful in their operations, use arsenic. But to place a pill of arsenic in the tooth, destroying its sensibility for a time, and then filling immediately upon that mass of impurity and cause of disease, is a thing which I objected to then and object to now; but having had the manner explained to me by Dr. Maynard, I have had success in using it in that way. But we all agree that excision is decidedly the most successful method, and that arsenic should never be used, unless the whole portion so destroyed, is removed afterwards, and the cavity of the tooth completely filled. I wished to make this explanation, that there might not be an erroneous impression abroad.

Dr. Westcott.—I would like to make a similar explanation. I think there must have been some mistake in regard to the general sweeping assertion that Dr. Bridges said I made. It is true that, as I then look upon it, it was, to say the least of it, generally in quacks' hands. I think, if Dr. Bridges will recall the circumstances, he will modify it. My temperament, I know, is rather an ardent one, but I have no remembrance of ever saying that a man could not possibly use a certain thing without being a quack. That is going a good ways. But I am ready to say that the head and front of all the quackery that comes to my knowledge is from the use of arsenic. I look upon it as detestable under all circumstances where the object is merely to destroy the sensibility of the tooth; but the precautions and means are so much better understood now than when the remark was made, that I think it is comparatively safe.

Dr. Arthur.—I desire that a remark of the President may be so modified as not to leave the impression that he was giving the views of the society. He said, "we all agree that excision," without the previous use of any substance to destroy the vitality of the pulp, "is the most successful method."

The President.—I did not mean to convey the idea that we, as a society, but that Dr. Dunning and myself thought so.

Dr. J. Parmly.—Every one here will admit that there is a good and a bad way of doing things. The arsenic may be applied so as to do great good, and it may be applied so as to do great harm. I have seen cases where the nerve was in a highly inflamed state at the time, and then a pill of arsenic was put in and a piece of cotton put over it; and perhaps after a few days this would be removed and a dry piece put in, and then the tooth would be filled with gold or amalgam over that cotton, leaving a mass of impurity in the cavity which would, in six months, turn the tooth to a dark color, and it was evidently dead. Who will not perceive the difference between that kind of practice and simply putting in for a few days this application of arsenic, and then removing it and all the soft part and filling it with gold? That has been my experience and observation in the use of arsenic—that there was a good and a bad way of using it;—one is decidedly bad and the other is not so objectionable. I don't think there is so much in the tooth as in the dentist. In regard to the first aphorism, I understand that in a certain state of the tooth, where the nerve is slightly exposed, it is better to treat it in that way. I do not understand that it is better to drill into every nerve and take it out, when the nerve is in a healthy state. I do not see how any one can understand it so. All I can say upon this subject comes from observation and not from practice, for I practice but very little in that way; but I have had a chance of seeing cases where Dr. Dunning has performed this operation—has taken out the nerve, and in fifteen minutes had the gold put into the fang of the tooth; and I have been more surprised to know how he got the nerve out of both fangs, than to know how a squirrel gets the meat out of both sides of the hickory nut by making a hole in but one side. You could not perceive, afterwards, the least possible disease or discoloration about the teeth or gums. I have seen many such instances; and the cases he has treated by taking out the nerve directly, have been, invariably the most successful. I have never seen the same success in cases where he has applied the arsenic and afterward filled the tooth. Six or seven years ago, I should have said that it was a very hazardous operation to take the nerve right out and put the gold in; but from seeing it done so often, I should now say it is the most successful way, and I would have a tooth of mine treated in that way. I have a tooth in my own mouth that was treated by Dr. Foster, twelve years ago. He came very near the nerve, and I told him if he came to it to pick it out, or to take the tooth out. He came so near it that it could be, absolutely, seen. He put over it a cap, and filled it with gold. I had the filling in my tooth for over ten years. And you will recollect, Mr. Chairman, that I was very anxious to have the tooth extracted, as I was under the impression that the nerve was dying. But I was persuaded to leave it; and I think about two years ago the filling broke out. At the time I did not feel any unpleasant sensation or taste, as I expected there would be when the cap gave way. On returning to New York, Dr. Dunning examined the tooth, and found that there was a perfect formation of the bone. He filled the tooth without touching the nerve and without giving me any pain, and I still have the tooth. The nerve was living at the time. This is all I have to state upon this subject. I rose merely for the purpose of saying that I thought the aphorisms would be generally understood.

[To be continued.]

